# Manual therapy interventions in the treatment of plantar fasciitis: A comparison of three approaches

**DOI:** 10.4102/hsag.v24i0.1244

**Published:** 2019-09-25

**Authors:** Christopher Yelverton, Sunil Rama, Bernhard Zipfel

**Affiliations:** 1 Department of Chiropractic, University of Johannesburg, Johannesburg, South Africa; 2 Evolutionary Studies Institute, University of the Witwatersrand, Witwatersrand, South Africa

**Keywords:** Chiropractic, McGill Pain Questionnaire and Foot Functional Index, Goniometer, Algometer, Plantar Fasciitis

## Abstract

**Background:**

Plantar fasciitis is one of the common causes of heel pain and a common musculoskeletal problem often observed by clinicians. Numerous options are available in treating plantar fasciitis conservatively, but no previous studies have compared combined conservative management protocols.

**Aim:**

The aim of this study was to compare manipulation of the foot and ankle and cross friction massage of the plantar fascia; cross friction massage of the plantar fascia and gastrocsoleus complex stretching; and a combination of the aforementioned protocols in the treatment of plantar fasciitis.

**Setting:**

This study was conducted at the University of Johannesburg, Chiropractic Day Clinic, and included participants that complied with relevant inclusion criteria.

**Methods:**

Forty-five participants between the ages of 18 and 50 years with heel pain for more than 3 months were divided into three groups and received one of the proposed treatment interventions. The data collected were range of motion (ROM) of the ankle (using a goniometer) and pain perception using the McGill Pain Questionnaire and Functional foot index and algometer.

**Results:**

The results of this study indicate that cross friction massage of the plantar fascia and stretching of the gastrocsoleus complex showed the greatest overall improvement in terms of reducing the pain and disability and ankle dorsiflexion ROM, whereas the combination group showed the greatest increase in plantar flexion.

**Conclusion:**

The results demonstrated that all three protocols had a positive effect on the ROM and pain perception to patients with plantar fasciitis.

## Introduction

### Background

Plantar fasciitis is a common cause of heel pain (Baravarian 2009; Buchbinder 2004; Schwartz & Su 2014; Young, Rutherford & Niedfeldt 2001) and a common musculoskeletal problem. The condition is defined as a sharp pain at the medial plantar aspect of the calcaneus and medial longitudinal arch of the foot. It is usually aggravated with the first few steps in the morning and after long periods of non-weight-bearing that diminishes as walking progresses, with fascial stretching and metabolite dispersing (Barrett & O’Malley 1999; Buchbinder 2004; Dubin 2007; Roxas 2005; Schwartz & Su 2014; Young et al. 2001).

Although research has been conducted in the conservative treatment of plantar fasciitis, sporting persons, children and even inactive individuals are still vulnerable to this condition owing to its multifactorial aetiology (Lynch et al. 1998; Roxas 2005), which include anatomical, biomechanical and environmental factors. It follows that a lack of understanding of the biomechanics of plantar fasciitis may result in an inadequate treatment plan. Numerous options are available in treating plantar fasciitis, with conservative measures frequently documented (Baravarian 2009; Dubin 2007; Young et al. 2004). These measures include stretching, orthoses, advice on weight loss, night splints, physical therapy modalities, anti-inflammatory agents (such as corticosteroid injections), surgery and protein-rich plasma (Akşahin et al. 2012; Buchbinder 2004; Goff & Crawford 2011; Lynch et al. 1998; Martin et al. 2001; Powell et al. 1998; Schepsis, Leach & Gouyca 1991). Ninety per cent of patients should respond to conservative therapy, and only those with chronic presentations (over 6 months) should consider extracorporeal shockwave therapy or plantar fasciotomy, or endoscopic plantar release (Cutts et al. 2015; Goff & Crawford 2011).

### Pathology of plantar fasciitis

Although a common condition, the exact mechanism and aetiology is not fully understood, but is generally accepted to be associated with repetitive microtrauma (Cutts et al. 2015).

**FIGURE 1:**
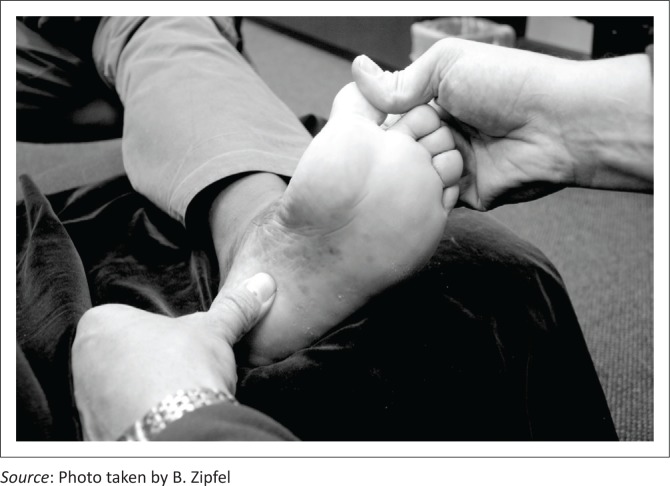
Foot with the hallux hyper-dorsiflexed placing tension on the medial band of the plantar fascia and calcaneal enthesis.

Inflammation may cause an enthesopathy or fasciosis at the calcaneal insertion, which is responsible for much of the pain and discomfort (Cornwall & McPoil 1999; Schwartz & Su 2014). The location of pain in the heel region may vary, with pain often being reported over the medial, lateral and lower posterior aspects of the calcaneus and the inferior heel region (inside part of the heel), most typically the medial plantar process (Cornwall & McPoil 1999) (Figure 1). On occasion, the patient may also complain of pain over the central band of the plantar fascia in the region of the medial longitudinal arch. Typically, the pain is most significant during weight-bearing activities and, in most cases, there has been a change in either the amount or intensity of physical activity before the onset of the patient’s symptoms (Kosmahl & Kosmahl 1987; Schwartz & Su 2014).

Here we present three manual therapy approaches, both singularly and in combination.

### Methods of management utilised in this study

Cross friction massage, or transverse massage, is a technique applied over an area of trauma or inflammation to reduce adhesions and prevent excessive scar tissue formation. This technique induces a traumatic hyperaemia, facilitating the elimination of substance P, which is achieved by the release of histamine (Cassar 2004). Soft tissue techniques that stretch the plantar fascia, and progressive cross friction massage at the origin of the fascia to break down the scar tissue, may improve plantar fascia flexibility. This may be enhanced by joint mobilisations (which mobilise the rearfoot, subtalar joint and navicular) (Hertling & Kessler 2006). This technique has been shown to be beneficial in inflammatory tendon conditions, resulting in a breakdown of adhesions and creating new collagen fibres to replace the immature collagen found in tendinosis (Digiovanni et al. 2006; Hammer 1999), thus facilitating the repair process. In conjunction with stretching exercises, it is proposed to promote remodelling of the injured tissue along its correct lines, thereby increasing flexibility and reducing pain (Schwartz & Su 2014).

Stretching of the triceps surae and plantar fascia has been shown to improve the range of motion (ROM) of the talocrural joint in dorsiflexion and facilitate the treatment of plantar fasciitis (Dubin 2007; Schwartz & Su 2014).

Manipulation of restricted joints in the ankle may produce more motion and enhanced function at that joint, which is thought to be compromised in plantar fasciitis (Dubin 2007). Polkinghorn (1995) found that conservative management utilising chiropractic manipulation may be effective, and recommended a trial period of manipulative therapy in cases of plantar fasciitis. Brantingham et al. (1992) and Dimou, Brantingham and Wood (2004) concluded that chiropractic manipulation is an effective treatment for plantar fasciitis.

### Aim of the study

We carried out a study to determine the protocol that would yield the greatest reduction in pain and improved functional movement. The three treatment protocols utilised were foot and ankle manipulation combined with cross friction massage; gastrocsoleus complex stretching combined with cross friction massage; and a combination of foot and ankle manipulation, cross friction massage and gastrocsoleus complex stretching.

## Research method and design

### Study design

This study was designed as a prospective, quantitative, randomised, comparative study, with three different intervention groups.

### Participant recruitment and random group allocation

Participants were recruited by means of advertising posters that were placed around the University of Johannesburg Health Clinic and campus, and surrounding companies. E-mails were also sent out to running clubs. Any respondent to the advertisements was considered as a potential candidate for the study.

Following acceptance into the research study, each participant received an assessment to diagnose the condition of plantar fasciitis according to the inclusion criteria listed below. Forty-five participants (*n* = 45) were randomly allocated to three groups (*n* = 15 per group), by selection of numbers 1–3 and allocation to the relevant treatment intervention grouping.

### Inclusion criteria

To be included in the study, participants needed to comply with the following criteria consistent with ‘typical’ plantar fasciitis:

- Be between the ages of 18 and 50.
- The duration of pain had to be 6 weeks or more, categorising it into the chronic phase (Scioli 1994).
- Pain needed to be well localised to the medial calcaneal tuberosity (Young et al. 2001).
- Present with heel pain, described as worse in the morning upon contact with the ground, and decreasing as walking progresses.
- Pain needed to increase upon toe standing (Barrett & O’Malley 1999; Brown 1996; Pollard & So 1999; Reid 1992).
- There was restriction in motion palpation findings of the talocrural, subtalar and midtarsal joints.

### Exclusion criteria

Potential participants were excluded if they presented with the following:

- A history of hip, knee, foot or ankle surgery or stress fractures of the calcaneus.
- Received manual therapy, cortisone injection or used anti-inflammatory medication 3 weeks prior to or during the study.
- Pregnancy (because of changes in weight and potential pedal oedema which may result in heel pain).
- Conditions such as ankylosing spondylitis, systemic lupus erythematous, peripheral neuropathy, Sever’s disease and tarsal tunnel.

### Treatment interventions

Group 1 received mobilisation and manipulation to the ankle and foot with cross friction of the plantar fascia. Group 2 received stretching of the gastrocsoleus complex and cross friction of the plantar fascia. Group 3 received a combination of the three protocol, including mobilisation and manipulation of the foot and ankle, stretching of the gastrocsoleus complex and cross friction of the plantar fascia.

All participants received six visits over a 3-week period. This was staggered as three visits in week 1, two visits in week 2 and the final visit in week 3.

The following treatment interventions were utilised for this study.

#### Chiropractic manipulation

The following chiropractic manipulative techniques were utilised dependent on the joint restrictions found, as described by Kirk, Lawrence and Valvo (1985).

**Mortise separation:** The participant was placed supine with either hand of the researcher grasping the medial and lateral border of the foot with the thumb on the sole of the foot and fingers on the dorsum. The foot was then motioned through dorsiflexion, internal rotation and eversion with an impulse thrust delivered parallel to the researcher.

**Mortise shear:** The participant was placed supine with the involved knee in flexion. One hand was placed proximal to the mortise joint (tibio-talo joint) and the other hand was placed distal to the joint. The joint was then sheared in an anterior to posterior direction.

**Foot figure eight:** The participant was placed supine and one hand was placed on the lateral aspect of the ankle and calcaneus, holding it from underneath. The other hand was placed on the medial aspect of the midfoot with the thumb on the sole of the foot and the fingers on the dorsum. The ankle was kept stable, while the forefoot and midfoot was moved through a combination of inversion with abduction and eversion. The motion has a medial to lateral orientation (figure of eight).

**Metatarsal shear:** The participant was supine. The foot was grasped on either side with the thumbs placed on the metatarsal head on the sole of the foot. Each metatarsal head of the affected foot was translated back and forth.

**Hallux mobilisation technique:** The participant was supine and the researcher was at the foot of the table, with one hand stabilising the foot and the other hand grasped the patient’s hallux, mobilising it in all directions with a medial thrust.

**Tarsal–metatarsal shear:** The participant was placed supine with the knee flexed and the researcher at the side of the table, with one hand grasping the foot firmly, contacting just proximal to the tarsal–metatarsal joint. The foot was kept dorsiflexed. The other hand was placed firmly distal to the tarsal–metatarsal joint, and sheared the joints in a dorsal to plantar and backward direction while the other hand held the heel firmly on the ground.

**General calcaneal technique:** The participant was placed prone. The researcher stood at the foot of the table so that the sole of the patient’s foot was firmly fixed to the researcher’s abdomen. The calcaneus was grasped on either side with interlaced fingers and then moved through a circular motion.

#### Cross friction massage

Participants were placed in a supine position for this intervention. Lubricant was not used here, so that the finger (usually one, but sometimes two) doing the massage does not slide across the skin but rather takes the skin with it, allowing for the force to be transmitted directly to the deep tissue being treated. The most painful area of the plantar fascia insertion was located and the foot dorsiflexed to allow stretching of the plantar fascia. Deep friction massage at the insertion of the plantar fascia was then employed using a reinforced index finger. The motion was kept approximately within an inch, back and forth ‘across the grain’ of the tissue. The friction massage was sustained for 5 min (De Bruijn 1984). The amount of pressure used for and time spent on the technique was consistent in all groups and at every treatment.

#### Stretching protocols

**Gastrocnemius muscle stretch:** The participant was instructed to lean against a wall with both hands shoulder-width apart. The unaffected leg was brought forward and the affected leg was taken backwards. The knee of the front leg was flexed and the knee of the back leg was kept extended. The heels of both feet were kept on the ground throughout the stretch. The participant then leant forward until a maximal stretch was felt in the calf area. The protocol for every participant was a 30-s stretch, 3 times a day for the 3-week period.

**Soleus muscle stretching:** The participant was instructed to lean against a wall with both hands shoulder-width apart. The unaffected leg was brought forward and the affected leg was brought backwards. The knees of both legs were slightly flexed. The heels of both feet were kept on the ground throughout the stretch. The participant then leaned forward until a maximal stretch was felt in the calf area. The protocol for every participant was a 30-s stretch, 3 times a day for the 3-week period (Lorish, Thorsteinsson & Howard 1989). This protocol was thoroughly explained and each participant in groups 2 and 3 was carefully instructed.

### Data collection method

Data were obtained at visits 1 (baseline), 3, 5 and 6. All measurements occurred prior to treatment interventions.

The following measures were used to obtain information of changes in time for the treatment period.

#### Short-form McGill Pain Questionnaire

The short-form McGill Pain Questionnaire is a reliable measure of pain (Grafton & Kate 2005; Melzack 1985, 1987) and provides information regarding the perception and extent of pain. This questionnaire has two sections, including the sensory and afferent dimensions of pain. The questionnaire consists of 15 words that assess the patient’s perception of pain. Each word has four options to choose from, with a minimum scoring of 0 (no pain at all) to a maximum scoring of 3 (severe pain) per question. The score was added and then divided by the total number of questions answered by the participant (higher scores indicated a worsening of the participant’s pain).

#### Foot Functional Index

The Foot Function Index (FFI) is a validated short and simple measure of foot pain and disability (Budiman-Mak, Conrad & Roach 1991; Saag et al. 1996). Although it was originally designed to assess the effect of foot orthoses on foot pathology in patients with rheumatoid arthritis, it has been suggested by its developers that it is not limited to this group of patients (Budiman-Mak et al. 1991).

Participants were asked to answer a series of questions, rating the level of their pain between 0 (no pain at all) and 10 (worst pain) for each activity. The score was totalled and divided by 120 (12 questions × 10). The score was then recorded as a percentage (Budiman-Mak et al. 1991).

#### Algometer

The algometer was used to evaluate plantar heel tenderness, with the unit of measurement in kg/cm². This instrument is used to measure the pressure required to elicit pain threshold (i.e. when pain is first experienced). The device has been tested in a variety of settings and on different tissues of the body and found to be valid and reliable (Fischer 1987; Haneline 2007).

In this study, the patient was positioned in a supine position with the legs fully extended and the origin of the plantar fascia at the medial calcaneal tubercle was palpated. The ankle and toes were passively dorsiflexed and the algometer applied at the medial plantar process. The algometer contact head was aligned perpendicularly to the skin and the pressure gradually increased until the participant reported pain (pain tolerance). This process was repeated 3 times in the same manner, and three measurements were recorded at the same point on the plantar fascia.

An average of the three readings was recorded. Higher algometer scores indicated greater pressure threshold, therefore less tenderness. Lower algometer scores indicated less pressure threshold, thus more tenderness.

#### Goniometer

A universal goniometer was used to evaluate ankle ROM in dorsiflexion and plantar flexion. Joint ROM can be reliably measured using the universal goniometer, when preferably the same therapist performs the repeated measures using a ‘rigid standardised measurement protocol’. In the clinical setting, the universal goniometer is the goniometer most frequently used to measure ROM of extremity joints (Clarkson 2006). Menadue et al. (2006) conducted a study to measure the reliability of goniometric methods of the ankle ROM. They concluded that intra-observer reliability was high to very high within a test session, and between-session reliability was highest for inversion ROM. For the purposes of this study, as per findings of Youdas, Bogard and Suman (1993) that indicated inter-rater reliability is low, the same measurements were performed by one of the researchers (S.R.) at each period.

### Data analysis

The Mann–Whitney *U* test (Wilcoxon) was used to compare two independent samples. The probability level or *p*-value for statistically significant differences was set at 0.05. Therefore, there is a statistically significant difference between the means of the groups compared if *p* ≤ 0.05. If *p* > 0.05, then a statistically significant difference does not exist between the means of the groups compared.

Groups were tested for normality to determine if parametric or non-parametric testing would be utilised on data. Dorsiflexion and algometer values were determined to be normally distributed and ANOVA was utilised, with Kruskal–Wallis and Mann–Whitney test for non-normally distributed values of plantar flexion, McGill Pain Questionnaire and FFI.

### Ethical consideration

This study was approved by the University of Johannesburg Ethics Committee (clearance number AEC24/2009).

## Results

### The McGill Pain Questionnaire

Group 1 began with a mean value score of 0.52 at measurement 1, which decreased to 0.33 at measurement 2, to 0.29 at measurement 3, with a further decrease to 0.24 at measurement 4. A statistically significant difference in pain perception was noted between measurements 1 and 2 (*p* = 0.001), with a mean difference of 0.19. There was also a statistically significant difference between measurements 1 and 4 (*p* = 0.001), with a mean difference of 0.28.

Group 2 began with a mean value score of 0.62 at measurement 1, which decreased to 0.47 at measurement 2, to 0.39 at measurement 3, with a further decrease to 0.25 at measurement 4. A statistically significant difference in pain perception between measurements 1 and 2 (*p* = 0.006) was noted, with a mean difference of 0.15. There was a statistically significant difference between measurements 1 and 3 (*p* = 0.030), with a mean value of 0.08; measurements 3 and 4 (*p* = 0.005), with a mean value of 0.14 and measurements 1 and 4 (*p* = 0.001), with a mean value of 0.37.

**FIGURE 2:**
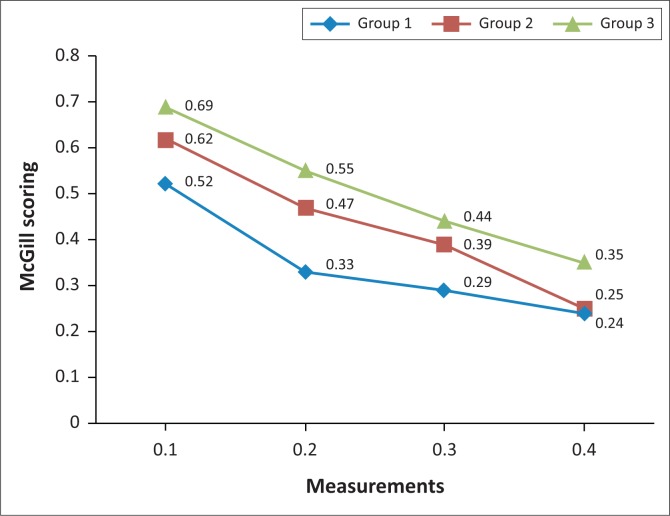
Line graph indicating McGill Pain Questionnaire measurements. Group 1 = mobilisation and manipulation to the ankle and foot with cross friction of plantar fascia; group 2 = stretching of gastrocsoleus complex and cross friction of plantar fascia; group 3 = combination of the three therapies.

Group 3 began with a mean value score of 0.69 at measurement 1, which decreased to 0.55 at measurement 2, to 0.44 at measurement 3, with a further decrease to 0.35 at measurement 4. No significant difference between measurements 1 and 2 was noted, and thus they had similar results. A statistically significant between measurements 2 and 3 (*p* = 0.002) was noted with a mean value of 0.11; measurements 3 and 4 (*p* = 0.015), with a mean value of 0.09 and measurements 1 and 4 (*p* = 0.005), with a mean value of 0.34. Improvements indicated are represented in Figure 2.

### Foot Function Index

Group 1 began with a mean value of 31.73 at measurement 1, which decreased to 23.55 at measurement 2, to 20.06 at measurement 3, with a further decrease to 16.92 at measurement 4. A statistically significant difference was noted between measurements 1 and 2 (*p* = 0.003), with a mean difference of 8.18; measurements 2 and 3 (*p* = 0.013), with a mean difference of 3.49 and measurements 1 and 4 (0.001), with a mean difference of 14.81.

Group 2 began with a mean value of 26.73 at measurement 1, which decreased to 19.32 at measurement 2, to 12.22 at measurement 3, with a further decrease to 6.94 at measurement 4. A statistically significant difference was noted between measurements 1 and 2 (*p* = 0.003), with a mean difference of 7.41; measurements 2 and 3 (*p* = 0.002), with a mean difference of 7.10; measurements 3 and 4 (*p* = 0.003), with a mean difference of 5.28 and measurements 1 and 4 (*p* = 0.001), with a mean difference of 19.79.

**FIGURE 3:**
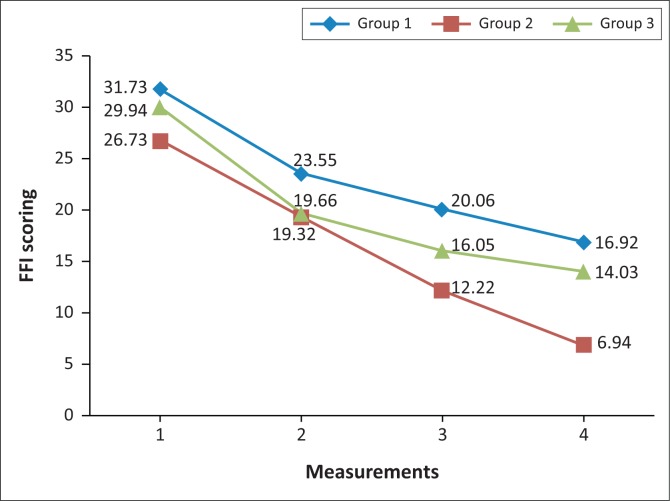
Line graph indicating FFI scoring. Group 1 = mobilisation and manipulation to the ankle and foot with cross friction of plantar fascia; group 2 = stretching of gastrocsoleus complex and cross friction of plantar fascia; group 3 = combination of the three therapies.

Group 3 began with a mean value of 29.94 at measurement 1, which decreased to 19.66 at measurement 2, to 16.05 at measurement 3, with a further decrease to 14.03 at measurement 4. A statistically significant difference was noted between measurements 1 and 2 (*p* = 0.003), with a mean difference of 10.28, and measurements 1 and 4 (*p* = 0.001), with a mean difference of 15.91. These measurements are illustrated in Figure 3.

### Ankle range of motion – dorsiflexion

Group 1 began with a mean value of 20.47° at measurement 1, which increased to 20.77° at measurement 2, to 21.43° at measurement 3, with a decrease to 21.40° at measurement 4. No statistically significant difference was noted in terms of mean value. However, the closest variable to being significant was between measurements 1 and 4 (*p* = 0.05).

Group 2 began with a mean value of 20.77° at measurement 1, which increased to 21.37° at measurement 2, to 22.17° at measurement 3, with a further increase to 23.60° at measurement four. A statistically significant difference was noted between measurements 3 and 4 (*p* = 0.002), with a mean difference of 1.43°, and measurements 1 and 4 (*p* = 0.001), with a mean difference of 2.83°.

Group 3 began with an initial mean value of 21.40° at measurement 1, which increased to 22.47° at measurement 2, decreased to 21.17° at measurement 3, with an increase to 23.50° at measurement 4. A statistically significant difference was measured between measurements 3 and 4 (*p* = 0.001), with a mean difference of 2.33°, and measurements 1 and 4 (*p* = 0.019), with a mean difference of 2.10°.

### Ankle range of motion – plantar flexion

Group 1 began with a mean value of 57.07° at measurement 1, which decreased to 55.97° at measurement 2, decreased to 55.67° at measurement 3 and increased to 56.13° at measurement 4. No statistically significant changes were noted.

Group 2 began with a mean value of 60.43° at measurement 1, which increased to 61.07° at measurement 2, to 64.00° at measurement 3, with a further increase to 64.87° at measurement 4. No statistically significant changes were noted.

**FIGURE 4:**
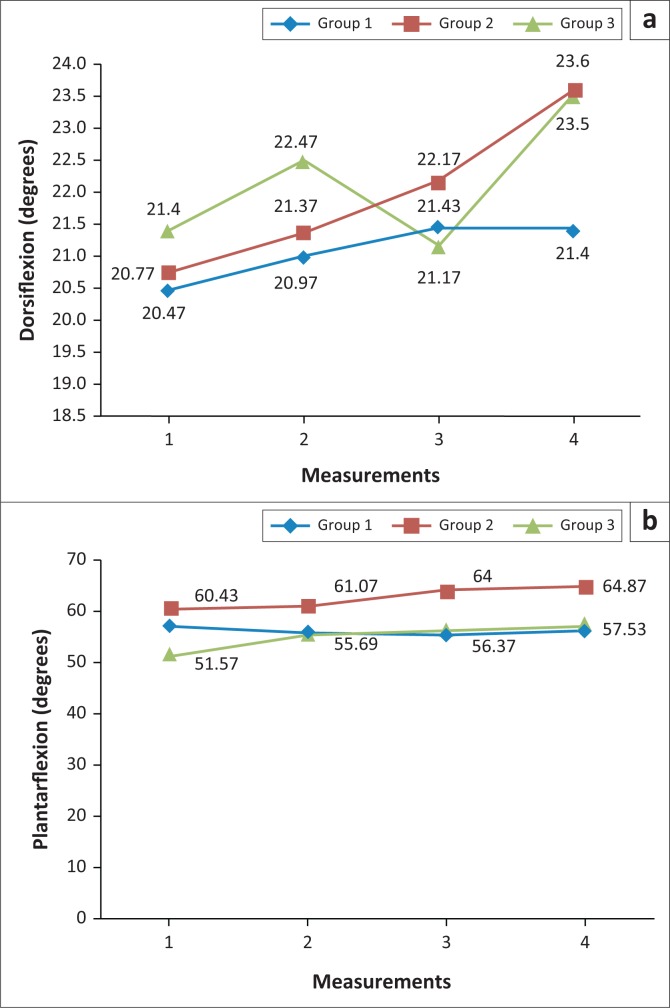
Line graphs indicating measurement changes for (a) dorsiflexion and (b) plantar flexion. Group 1 = mobilisation and manipulation to the ankle and foot with cross friction of plantar fascia; group 2 = stretching of gastrocsoleus complex and cross friction of plantar fascia; group 3 = combination of the three therapies.

Group 3 began with an initial mean value of 51.57° at measurement 1, which increased to 55.69° at measurement 2, to 56.37° at measurement 3, with a further increase to 57.53° at measurement 4. Statistically significant difference was noted between measurements 1 and 4 (*p* = 0.008), with a mean difference of 5.96°. Changes in ankle dorsiflexion and plantar flexion are represented in Figure 4.

### Algometer readings

Group 1 began with a mean value of 4.36 kg/cm² at measurement 1, which increased to 4.98 kg/cm² at measurement 2, to 5.18 kg/cm² at measurement 3, with a further increase to 5.34 kg/cm² at measurement 4. A statistically significant difference was noted between measurements 1 and 2 (*p* = 0.012), with a mean difference of 0.62 kg/cm², and measurements 1 and 4 (*p* = 0.001), with a mean difference of 0.98 kg/cm².

Group 2 began with a mean value of 4.42 kg/cm² at measurement 1, which decreased to 4.37 kg/cm² at measurement 2, to 5.62 kg/cm² at measurement 3, with a further increase to 6.25 kg/cm² at measurement 4. A statistically significant difference was measured between measurements 2 and 3 (*p* = 0.001), with a mean difference of 1.25 kg/cm²; measurements 3 and 4 (*p* = 0.006), with a mean difference of 0.63 kg/cm² and measurements 1 and 4 (*p* =0.001), with a mean difference of 1.83 kg/cm².

Group 3 began with an initial mean value of 4.35 kg/cm² at measurement 1, which increased to 5.16 kg/cm² at measurement 2, to 5.39 kg/cm² at measurement 3, with a further increase to 6.16 kg/cm² at measurement 4. A statistically significant difference was noted between measurements 1 and 2 (*p* = 0.001), with a mean difference of 0.81 kg/cm²; measurements 3 and 4 (*p* = 0.011), with a mean difference of 0.77 kg/cm² and measurements 1 and 4 (*p* = 0.001), with a mean difference of 1.81 kg/cm².

**FIGURE 5:**
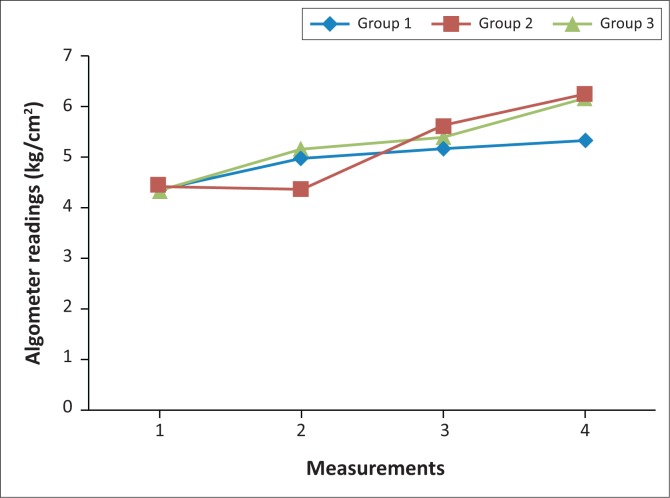
Line graph representing changes in algometer readings. Group 1 = mobilisation and manipulation to the ankle and foot with cross friction of plantar fascia; group 2 = stretching of gastrocsoleus complex and cross friction of plantar fascia; group 3 = combination of the three therapies.

Algometer reading changes are indicated in Figure 5.

### Intergroup analysis

ANOVA testing was utilised on dorsiflexion and algometer readings as they were normally distributed.

Dorsiflexion analysis revealed a statistically significant difference (*p* = 0.018) between groups, indicating that group 2 showed the greatest improvement.

Kruskal–Wallis testing revealed statistically significant differences in plantar flexion (*p* = 0.002) and FFI (*p* = 0.027), with group 2 showing the greatest improvement.

Mann–Whitney testing confirmed statistically significant differences between groups for dorsiflexion (*p* = 0.010), plantar flexion (*p* = 0.000) and FFI (*p* = 0.002), for group 2 when compared with group 1. This analysis also showed a statistically significant difference for plantar flexion on comparison between groups 2 and 3 (*p* = 0.20). No statistically significant changes were noted between groups 1 and 3.

## Discussion

Plantar fasciitis is classified as a syndrome that results from repeated trauma to the plantar fascia at its origin on the calcaneus resulting in pain and dysfunction and can often become a source of frustration to both the patient and clinician (Schwartz & Su 2014). To this end, we presented a number of effective non-invasive manual therapy protocols that may be used either on their own, or in conjunction with other treatments. Our research was concerned with determining which of the three protocols was the most effective in the treatment of plantar fasciitis. While all three interventions show an improvement, certain combinations of therapy showed more significant changes in certain measurements.

### The McGill Pain Questionnaire

There was a statistical difference in all three groups with regard to the McGill Pain Questionnaire, indicating in all intervention groups an improvement from the treatment.

### The Foot Function Index

There was a statistically significant difference in all three groups with regard to the FFI scoring, indicating all treatment approaches improved pain and disability. However, stretching and cross friction showed the greatest overall improvement in terms of reducing pain and disability.

### Dorsiflexion

Stretching combined with cross friction and the combination of all three treatments showed a statistically significant difference in dorsiflexion measurement over the treatment period. The stretching combined with cross friction showed statistically significant improvement compared with the other two interventions.

### Plantar flexion

There was a statistically significant difference for plantar flexion in the combination of all three treatments, with the stretching and cross friction showing statistically significant improvement compared with the other two approaches.

### Algometer

There was a statistically significant difference for algometer readings within all three groups, indicating all three approaches reduce pain threshold.

All three protocols included cross friction therapy to the insertion of the plantar fascia. The above findings all show decreased pain perception. It is proposed that pain reduction is as a result of central pain modulation and the pain control theory where type A delta and C nerve fibres are inhibited by stimulation of large diameter fibres in the substantia gelatinosa of the spinal cord. In addition, this approach decreases pain-producing metabolites and breaks down cross bridges or adhesion of the connective tissue (Hasan et al. 2016). In addition, this approach is proposed to accelerate healing at a physiologic level (Joseph et al. 2012). This would explain the improvements noted in the McGill Pain Questionnaire, Foot Function Index and algometer readings.

The stretching protocol, as described, included a dorsiflexion stretch to address proposed tightness in the gastrocnemius and soleus muscles. Passive stretching has been shown to increase ankle ROM by changing the muscle tendon unit (Nakamura et al. 2012). It would therefore be expected that increased ROM in the direction of passive stretch would occur. The combination of this stretching with cross friction seems to be the most advantageous for the patient in terms of overall effect. The pain reduction noted from cross friction may allow for greater ROM stretching, hence the improvement noted with the combination.

As proposed above, the effects of the treatments utilised can explain the changes noted. From the data obtained, it does not appear that one intervention allows for significant changes in the overall management of plantar fasciitis, with no synergistic effects of manipulation with the interventions (that may be expected based on the principles of pain control theory). Range of motion in plantar flexion was, however, improved significantly in the combination therapy, which could indicate a synergy between the approaches enhancing the effect over one alone.

## Conclusion

The results of this study suggest that all three of the approaches utilised have a beneficial effect in patients with plantar fasciitis. The use of manipulation seems to increase plantar flexion, while passive stretching increases ROM and decreases pain.

These findings may assist manual therapists and podiatrists in determining (based on the patient’s presentation) the combination of treatments that would effectively treat plantar fasciitis. Practitioners should approach patients in terms of their individual presentations and determine which combination of interventions suits their individual needs.
